# Posttraumatic Cortical Defect of Femur

**DOI:** 10.1155/2013/815460

**Published:** 2013-10-02

**Authors:** Jeyaseelan Nadarajah, Deep N. Srivastava, Rajesh Malhotra, Aravindh Palaniswamy

**Affiliations:** ^1^Department of Radiodiagnosis, All India Institute of Medical Science, New Delhi 110029, India; ^2^Department of Orthopaedics, All India Institute of Medical Science, New Delhi 110029, India

## Abstract

Posttraumatic cortical defect of bone is a rare entity which occurs in a maturing skeleton following green stick or torus fracture. Most of the cases are asymptomatic and they are detected incidentally on radiograph. These lesions usually require no treatment. However, the appearance of these lesions can mimic various pathological conditions affecting bone. Knowledge about this entity is important as it avoids unnecessary investigations. We present this case as the occurrence of this entity in femur is very rare and the child was symptomatic.

## 1. Introduction

Posttraumatic cortical defect of bone is a rare entity which occurs in a maturing skeleton following green stick or torus fracture [1]. They can occur in any long bone and are typically reported in distal radius and rarely in tibia and fibula [2]. To our knowledge, a single case report of occurrence of posttraumatic cortical defect in femur has been reported [3]. Most of the cases are asymptomatic and they are detected incidentally on followup radiograph. These lesions usually disappear in months to years without any treatment. Knowledge about this entity is important as it avoids unnecessary investigations. We present this case as the occurrence of this entity in femur is very rare and the child was symptomatic.

## 2. Case Report

**Figure 1:**
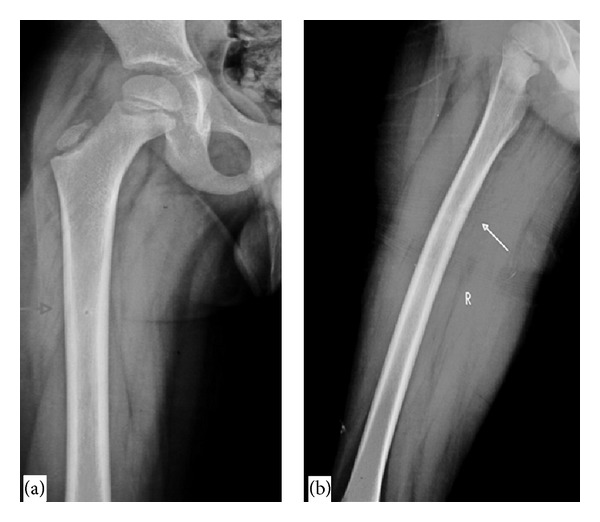
AP (a) and lateral (b) radiograph of the right femur show a well-defined linear radiolucent defect along the medial cortex of femur in its upper and middle third junction with sclerotic rim.

**Figure 2:**
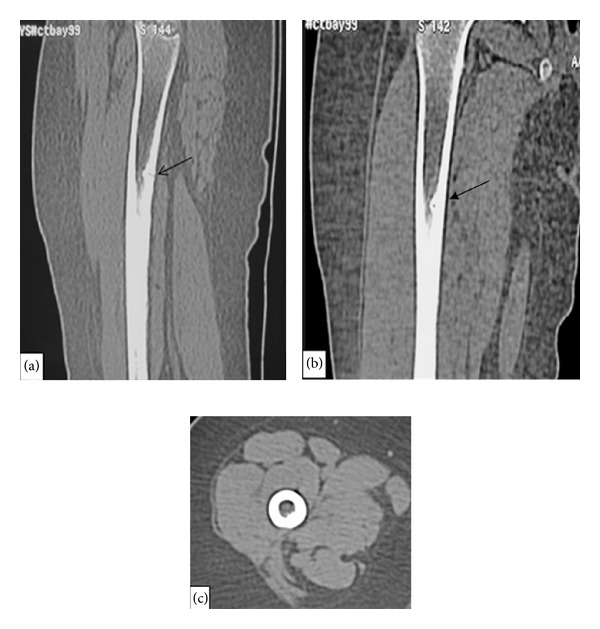
Sagittal (a), coronal (b), and axial (c) reconstructed CT images show a linear defect (arrow) in the medial cortex of the right femur with sclerotic surrounding.

**Figure 3:**
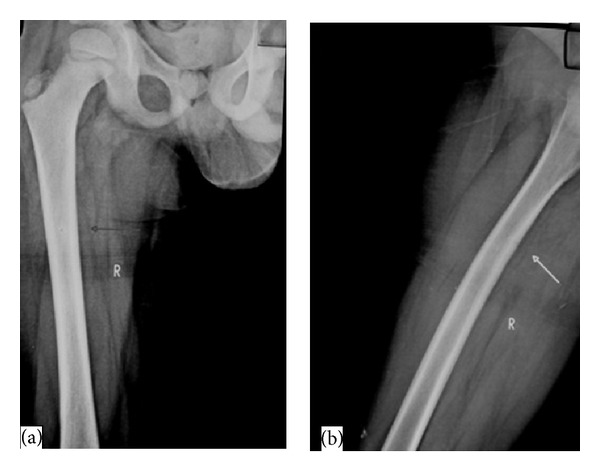
Plain radiograph AP (a) and lateral (b) views of the right femur done after 6 weeks show no significant change in the appearance of lucent bony defect noted along the medial cortex of the right femur.

A 5-year-old male child presented with a history of persistent dull aching pain following a fall during playing over the right mid thigh for 3 weeks. Clinical examination revealed no bony swelling over the thigh. Plain radiograph done immediately following trauma reported, suspicion of torus fracture of the proximal femur (not illustrated), for which he was placed in a plaster cast for 2 weeks. Plain radiograph was repeated after three months as the child complained of a dull aching pain which revealed a linear nonexpansile lucent cortical defect in posteromedial cortex of right femur in its upper third (Figure 1). Radiographs were repeated at 3 (not illustrated) and 6 (Figure 3) weeks later which revealed a persistent defect without significant change in its appearance. Based on these features, diagnosis of posttraumatic cortical defect was made. Follow up CT (Figure 2) was also done which confirmed the findings seen on plain radiographs. The patient was informed about submission of data for publication and informed consent was obtained prior to being included in the study.

## 3. Discussion

Green stick fracture is one of the common types of fractures that occur in an immature skeleton. Mechanism involves angular force that breeches one cortex without extension into the medullary cavity. These fractures are usually uncomplicated and they heal with immobilization. Development of cyst-like lucent defect near the fracture site can occur following the healing of green stick or torus fracture. All reported cases were detected incidentally during followup radiograph. The majority of the cases have been reported in radius and rarely in tibia, fibula, and femur. A typical lesion is nonexpansile, solitary, and sharply demarcated defect measuring approximately 10 mm in diameter and occurring within 10 mm of the compression point which resolves with time in months with tendency to diaphyseal migration. These lesions can be multiple, large, and expansile.

Etiology of this defect may be due to the inclusion of medullary fat within the subperiosteal haematoma, confirmed on CT and MRI [4, 5], or resorption cysts within an excessive periosteal reaction and subperiosteal hematoma [1, 6]. 

On the initial radiograph, the appearance of defect can mimic osteomyelitis, intraosseous ganglia, fibrous cortical defect, osseous involvement in neurofibromatosis, and even osteoid osteoma as it happened in our case as the child complained of pain. Careful clinical history, typical location, and serial followup radiographs are required to confirm the diagnosis. Though these defects are clinically insignificant, correct identification is required to avoid unnecessary investigation and counseling the parents.
